# Respect Your Elders: The Relation Between Generativity and Life Satisfaction in Custodial Grandparents

**DOI:** 10.1093/geroni/igaa057.1120

**Published:** 2020-12-16

**Authors:** Rachel Scott, Danielle Nadorff, Loriena Yancura, Melissa Barnett

**Affiliations:** 1 Mississippi State University, Starkville, Mississippi, United States; 2 Mississippi State University, Mississippi State, Mississippi, United States; 3 University of Hawai’i, Honolulu, Hawaii, United States; 4 The University of Arizona, Tucson, Arizona, United States

## Abstract

In Erikson’s (1950) theory of psychosocial development, generativity is defined as the drive to benefit future generations and leave a legacy. The prototypical individual satisfies this generative desire through parenthood in midlife. This stage has been shown to expand into later life due to grandparenthood (Erikson, 1982). Generativity has been shown to predict life satisfaction, but the amount of generative concern and action can be impacted by factors such as perceived respect from younger generations (Cheng, 2009). The current study examined adults aged 40 and older (M age = 61 yr) using a nationwide sample from Qualtrics Panel Service to assess the extent to which perceived respect moderates the relation between generativity and life satisfaction in custodial grandparents. Results indicated that perceived respect from the grandchild was found to have a moderating effect on the relation between generativity of custodial grandparents and life satisfaction. Results suggest that for those who perceive low levels of respect from their grandchildren, the more generativity they express, the lower their life satisfaction. For those who perceive higher levels of respect from their grandchildren, the more generativity they express, the higher their life satisfaction. These findings suggest that as attempts to be generative increase, life satisfaction fluctuates. This may in turn impact the likelihood of generative actions from the custodial grandparent.

